# Enhancement of intracellular γ-tocopherol levels in cytokine-stimulated C3H 10T1/2 fibroblasts: relation to NO synthesis, isoprostane formation, and tocopherol oxidation

**DOI:** 10.1186/1472-6769-7-2

**Published:** 2007-07-03

**Authors:** Yuichiro Tanaka, Leslie A Lesoon Wood, Robert V Cooney

**Affiliations:** 1 University of Hawaii Cancer Research Center, Natural Products and Cancer Biology Program, 1236 Lauhala Street, Honolulu 96813, Hawaii

## Abstract

**Background:**

Stimulation of C3H 10T1/2 murine fibroblasts with interferon-γ(IFN) and bacterial lipopolysaccharide (LPS) generates reactive oxygen and nitrogen species leading to DNA damage, lipid oxidation, and tocopherol oxidation. The tocopherols possess unique chemical and biological properties that suggest they have important roles related to intracellular defense against radical-mediated damage.

**Results:**

Despite increased levels of reactive oxidants and decreased media tocopherol, cellular levels of γ-tocopherol, but not α-tocopherol, were observed to increase significantly when cells were treated with IFN/LPS. Inhibition of nitric oxide (NO) synthesis by a specific inhibitor of inducible NO synthase (iNOS) increased both intracellular α-tocopherol and γ-tocopherol concentrations, but did not significantly alter the reduction in media tocopherol levels caused by IFN/LPS treatment. Both exposure to exogenous NO and cellular synthesis of NO in cell culture increased media levels of 8-epi-prostaglandin F2α, a marker of oxidative lipid damage, whereas inhibition of endogenous NO synthesis reduced media 8-epi-prostaglandin F2α formation to control levels.

**Conclusion:**

Elevated intracellular levels of γ-tocopherol in response to the cellular inflammatory state may indicate that it serves a unique role in minimizing cellular damage resulting from endogenous NO synthesis. Results of the current study suggest that NO is an important mediator of damage within the cell, as well as in the oxidation of both α- and γ-tocopherols. The paradoxical increase in cellular tocopherol associated with the induction of NO synthesis may indicate either enhanced cellular transport/decreased export for tocopherols or recruitment of free tocopherol from tocopherol storage molecules.

## Background

Vitamin E, a term used to describe the family of related tocopherol and tocotrienol species of widely varying bioactivity, was discovered over eighty years ago by Evans and Bishop [1]. The vitamin E bioactivity of tocopherols is characterized by their ability to prevent certain reproductive abnormalities, muscle wasting, and red blood cell fragility in mammals [2]. Dietary levels of vitamin E needed to meet these essential functions are relatively modest, are generally provided for from a balanced diet, and are principally associated with α-tocopherol, the predominant form of tocopherol found in plasma [2]. Recently, roles for the tocopherols in human and animal health, beyond those traditionally associated with vitamin E bioactivity, have been identified from epidemiological, clinical and basic laboratory research, suggesting that tocopherols may modulate the development and progression of cardiovascular disease [3,4], cancer [5,6] and neurological abnormalities [2,7], as well as affect immune function [8], natriuresis [9], and inflammation [10]. These newly identified functions of the tocopherols appear un-related to their vitamin E associated bioactivity, rather, it is observed that subtle chemical differences in structure and/or function between the various tocopherols are responsible for the different biological properties they manifest [11-13]. Indeed, these properties may be important in explaining differential epidemiologic associations observed for α- and γ-tocopherols with cancer incidence [6,14-16], and suggest unique mechanisms for tocopherol function, distinct from those traditionally associated with vitamin E bioactivity. These alternative tocopherol functions are often observed at relatively elevated doses (20 – 100 μM) in cell culture and include effects on cellular proliferation, apoptosis, cellular adhesion, regulation of gene expression, inflammation, and cellular signaling [reviewed in 17, 18] that logically could have effects on many aging-related diseases. However, the absence of definitive evidence from human clinical trials that higher doses of tocopherols have beneficial long-term effects has precluded dietary recommendations for either higher amounts of α-tocopherol in the diet or the intake of other tocopherol analogues [2].

While γ-tocopherol is the main tocopherol in the U.S. diet, α-tocopherol predominates in human plasma, approximately 30 μM for α-tocopherol vs 5 μM for the γ-form [19]. It now appears that the relatively lower contribution of γ-tocopherol to plasma total tocopherol is due in part to its metabolism to form γ-carboxyethyl-hydroxychroman, a compound with natriuretic [9] and anti-inflammatory properties [10,20], as well as to its enhanced uptake by cells [21,22]. Because of its metabolism, it is more difficult to maintain plasma levels of γ-tocopherol sufficient to meet vitamin E requirements, however, if the metabolic pathway is blocked [23], plasma γ-tocopherol levels rise and γ-tocopherol is then equivalent to α-tocopherol in meeting the vitamin E requirement of rats [24]. The most recent dietary recommendations have established 15 mg/day of d-α-tocopherol as the requirement for humans, do not recognize any contribution from γ-tocopherol with respect to vitamin E bioactivity, and do not establish any minimum requirement for γ-tocopherol, as no unequivocal long-term or short-term need has been established [2]. While the official recommendation is restricted to α-tocopherol, based on currently accepted endpoints, it is unclear whether α-tocopherol is able to fully substitute for the potential functions of γ-tocopherol postulated from observed differences in the chemical and biological effects of the two analogues. However, in the case of human natriuretic factor, it is clear that enzymatic generation of the active metabolite preferentially occurs only with γ-tocopherol.

Although both α- and γ-tocopherols are effective anti-oxidants, each possesses unique chemical properties that offer advantages depending on the nature of the oxidant and the physical environment in which the oxidation reaction takes place. In general, it appears that although α-tocopherol is the predominant form responsible for quenching oxygen radical damage [25-27], γ-tocopherol is more effective against nucleophilic oxidants such as peroxynitrite [28] and nitrogen radicals such as NO_2 _[11,12]. In addition tocopherol oxidation products, such as tocopheryl quinones [29] and nitrite esters [11] have been implicated as potential DNA damaging agents and long-term exposure, even to small quantiies may limit the optimal beneficial level of tocopherols *in vivo *[30].

Reactive nitrogen species have been implicated in the generation of cellular damage associated with chronic inflammation [31]. NO, produced via the enzymatic conversion of arginine to citrulline by inducible NO synthase (iNOS) can easily oxidize to more reactive nitrogen species in the lipid environment of the cell resulting in enhanced potential for DNA damage [32]. iNOS inhibitors [33] and γ-tocopherol [11] have been shown to effectively block neoplastic transformation of C3H 10T1/2 cells in a standard carcinogenesis assay. Epidemiologic and clinical studies support a role for the tocopherols in the prevention of cancer, especially for prostate cancer [5,6,16,34]. The role of chronic inflammation, associated with cytokine and oxidant generation has been generally implicated in the process of cancer formation and progression [35] and NO may be an important mediator, both positively and negatively in this process.

α-Tocopherol is also known to enhance the immune response of vertebrates [36-38], facilitating the elimination of infectious organisms [39,40]. The effect of α-tocopherol on immune function increases as a function of serum tocopherol, well beyond levels necessary for optimal vitamin E bioactivity, as defined by traditional bioassays for vitamin E activity and recommended levels of dietary intake, suggesting a possible alternative mechanism of action. One of the key cellular responses to an invading pathogen is up-regulation of iNOS by many cell types, including macrophages, resulting in significant generation of NO [41]. In rodent macrophages and fibroblasts, this is often mediated by cytokines such as interferon-γ (IFN) coincident with exposure to bacterial lipopolysaccharides (LPS) or viruses, and the subsequent immune response is enhanced by α-tocopherol [42]. We have shown previously that γ-tocopherol, but not α-tocopherol, enhances cytokine-mediated NO synthesis in C3H 10T1/2 cells [43] and that γ-tocopherol more effectively protects against cell death in rat insulinoma cells stimulated to produce NO by interleukin 1-β [12]. A specific role for γ-tocopherol in immune function has not been established, however. We hypothesize that γ-tocopherol facilitates an enhanced immune response to infection by reducing NO-mediated intracellular damage, through its antioxidant effects, thereby allowing a greater oxidative attack toward a pathogen by cells. Using C3H 10T1/2 murine fibroblasts treated with IFN/LPS as a model for a cellular inflammatory response, we endeavored to determine the effect of endogenous NO formation on cellular tocopherol levels, as well as oxidation products of tocopherols and other key cellular biomolecules formed in cells and in the surrounding aqueous medium in an effort to discern the nature of the reactions occurring in the cellular environments in which the tocopherols function.

## Results

### Modulation of media and cellular tocopherol levels

**Figure 1:**
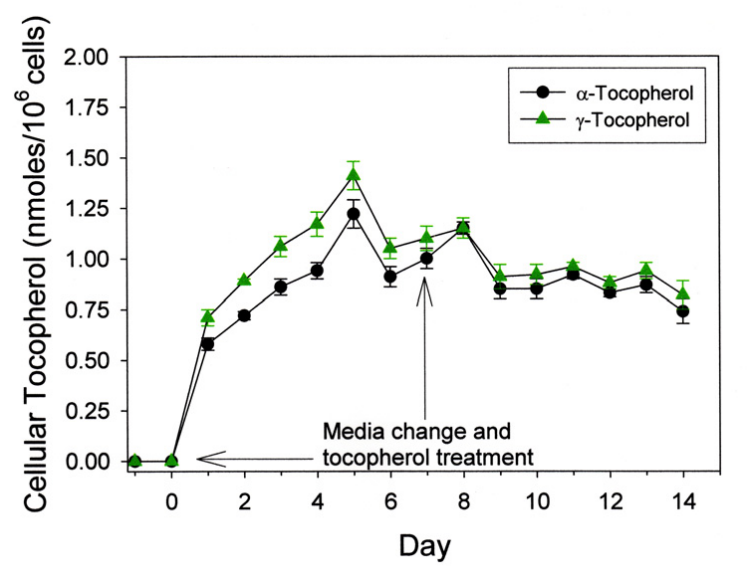
Cellular uptake of tocopherols treated with a mixture of tocopherols in medium. Cells were grown to confluence and given a media change followed by tocopherol treatment (Day 0). A second media change and tocopherol re-treatment was performed on Day 7. Tocopherols (α-,γ-; 5.0 μM each in ethanol) were measured in cells in triplicate and cell numbers for each treatment group determined as described under Methods for the extraction and analysis of tocopherols from cells and for cell counting. Data points represent the mean of three determinations ± SEM.

Preliminary experiments designed to study simultaneous uptake of alpha and gamma tocopherol analogues (5.0 μM each) indicated that tocopherol levels in C3H 10T1/2 cells were stable after seven days with re-treatment (Figure 1) and that no detectable levels of tocopherol were present in the culture medium in the absence of exogenous treatment. Loss in media tocopherol over this 14 day period was 29.5% and 17.9% for alpha- and gamma- tocopherols respectively. For both tocopherols peak cellular tocopherol levels were approximately 3–4% of the total tocopherol initially available to cells indicating that a significant fraction of α-tocopherol and γ-tocopherol could not be accounted for in the combined media and cellular analysis. It was also observed that cellular tocopherol levels increased as a function of external tocopherol concentration over the range of 0–100 μM tocopherol (data not shown).

Subsequent experiments exploring the role of oxidation in tocopherol uptake and metabolism used a protocol in which cells were pre-loaded with tocopherol for one week prior to treatment with IFN/LPS in order to assure stable cellular tocopherol levels. As shown in Figure 2A, cells treated with a combination of α- and γ-tocopherols (10 μM each) and IFN/LPS showed a significant decrease in media levels for both α- and γ-tocopherols relative to controls of approximately 15% over a 7-day period. This decrease in α- and γ-tocopherols in media was slightly, but not significantly, reversed by simultaneous inhibition of NO synthesis with 10 μM PBIT, a highly active and specific inhibitor of the iNOS protein. Treatment with PBIT alone slightly increased the observed media tocopherol levels over control values. In contrast, IFN/LPS treatment caused significantly increased cellular γ-tocopherol levels and slightly decreased α-tocopherol levels in cells (Figure 2B). PBIT treatment, however, enhanced intracellular levels of α- and γ-tocopherols in both IFN/LPS-treated and control cells (Figure 2B). This suggests that, in contrast to media, NO may mediate intracellular oxidation of α-tocopherol and γ-tocopherol. As seen in Table 1, the ratio of γ-/α-tocopherol is significantly elevated in cells relative to controls for all treatment groups, particularly in those treated with IFN/LPS alone. The observed change in ratio is particularly significant in that one tocopherol serves as an internal control for the other, and this ratio therefore is independent of any potential measurement error affecting the absolute values reported. Absolute values of α- and γ-tocopherols increased in both media and cells when NO production was blocked with PBIT, however, IFN/LPS treatment alters the tocopherol ratio in cells in favor of γ-tocopherol, independently of NO synthesis. Total recovery of tocopherol (media + cells) ranged from a low of 61.3% for α-tocopherol in cells treated with IFN/LPS to a high of 83.6% for γ-tocopherol in PBIT-treated cells (Table 1).

**Table 1:** Percent recovery and the ratio of γ-/α-tocopherol in the media fraction and cellular fraction of cells treated with IFN/LPS and/or PBIT as described in Figure 1. Values represent the mean of 6 determinations ± SEM.

	Tocopherol Recovery^# ^(%)			Ratio (γ/α tocopherol)		
Treatment	Media	Cells	Total	Media	Cells	Media vs Cells *p Value
	αT|γT	αT|γT	αT|γT			

Control	72.8|73.5	2.79|3.26	75.6|76.8	1.01 ± 0.01	1.17 ± 0.04	0.002
IFN/LPS	58.5|60.4	2.83|4.43	61.3|64.8	1.03 ± 0.01	1.57** ± 0.01	< 0.0001
IFN/LPS + PBIT	63.1|62.7	5.22|7.45	68.3|70.2	1.00 ± 0.01	1.43** ± 0.02	< 0.0001
PBIT	76.5|78.4	4.10|5.24	80.6|83.6	1.02 ± 0.01	1.27 ± 0.02	< 0.0001

* Student t-test for difference between media and cells.

** ANOVA for differences between treatment groups of log transformed values p < 0.01 relative to control cells.

# Based on treatment of 100 nmoles of each tocopherol per culture dish

**Figure 2:**
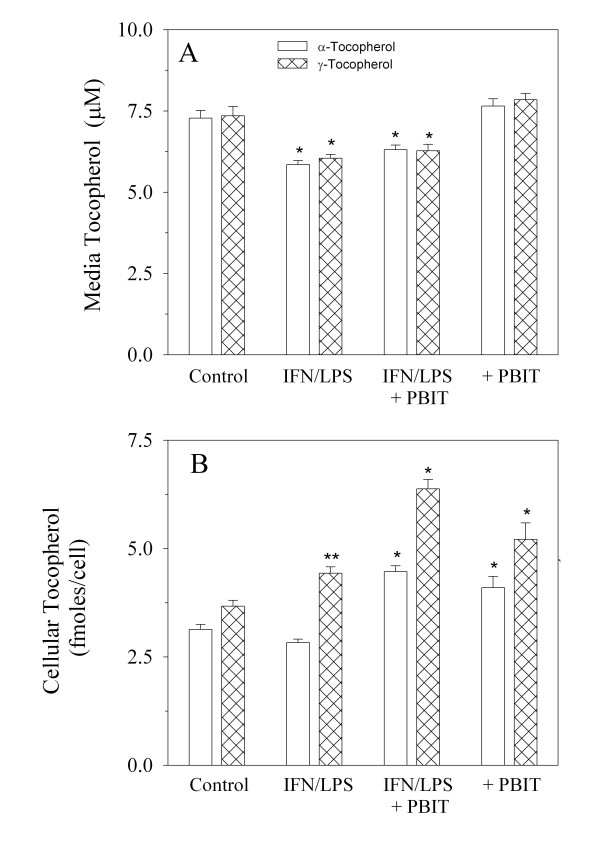
Effect of cellular NO synthesis on media and cellular levels of α- and γ-tocopherols. At confluence, a mixture of α-plus γ-tocopherol in 50 μl ethanol (10 μM final concentration/100 ng/dish for each tocopherol) was added to each 100 mm culture dish (10 mL of culture medium per dish). After seven days cell culture medium was changed and cells were re-treated with tocopherols. At this time six of the 12 dishes were also treated with IFN (10 ng/ml) & LPS (1 μg/ml) to stimulate NO production. Three culture plates that were treated with IFN/LPS and three that contained only the mixture of tocopherols, were also treated with 50 μl of 2 mM PBIT in PBS, yielding a final media concentration of 10 μM PBIT. The other 6 dishes were treated with either 50 μl PBS vehicle or 50 μl of 2 mM PBIT. Seven days later, media nitrite and 8-epi-prostaglandin F2α levels were measured as described in the methods section in duplicate. Tocopherol levels were measured in the cell culture medium (A) and in cells (B). Values represent the mean of six determinations ± SEM. * (p < 0.01) **(p < 0.05) Indicates significantly different from corresponding control value by ANOVA analysis. In addition all cellular γ-tocopherol levels were significantly higher than the corresponding α-tocopherol level for each treatment by student t-test (p < 0.01). PBIT treatment significantly elevated both α- and γ-tocopherols in IFN/LPS-treated cells (p < 0.01 by ANOVA).

### Role of nitric oxide in tocopherol oxidation

**Figure 3:**
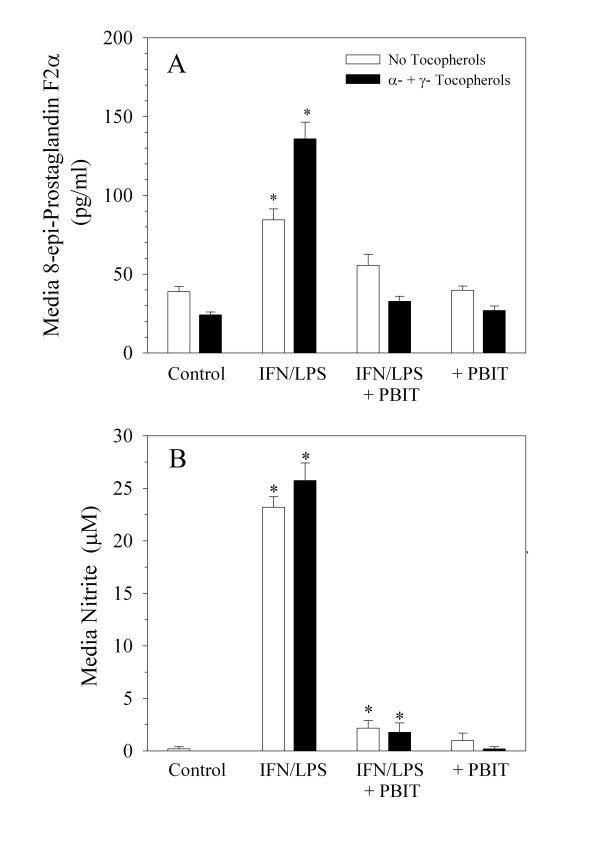
NO synthesis and its effect on isoprostane formation. Confluent cultures of C3H 10T1/2 cells were treated for seven days as indicated with a combination of 10 μM α-tocopherol and 10 μM γ-tocopherol or with 25 μL ethanol as a control in 35 mm culture dishes (5 ml total media volume per dish). The cell culture media was then changed and cultures were treated with PBS (control), IFN/LPS, and/or 10 μM PBIT as indicated ± tocopherols. After seven days, cell culture media was assayed for 8-epi-prostaglandin F2α (A) or nitrite (B) as described in Methods. Values reported represent the mean ± SEM (N = 9). * p < 0.01 relative to corresponding control (ANOVA analysis of log-transformed values). Tocopherol-treated isoprostane levels are significantly different for each treatment group in comparison to non-tocopherol-treated cells (p < 0.01). Nitrite values between tocopherol-treated and control cells were not significantly different.

**Figure 4:**
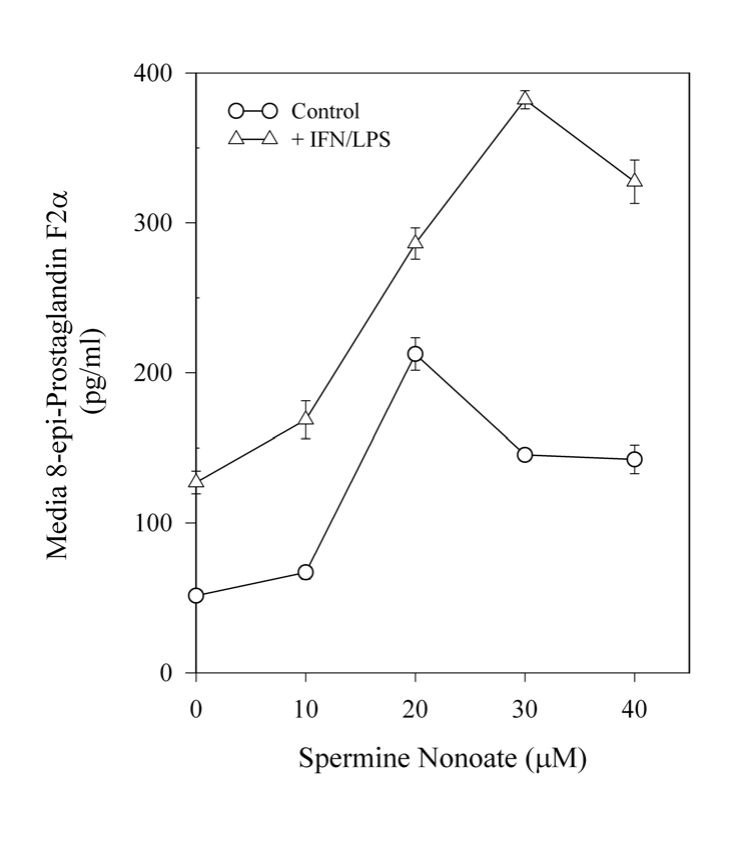
Formation of 8-epi-prostaglandin F2α by exogenous treatment of C3H 10T1/2 cells with spermine nonoate. Confluent cultures of C3H 10T1/2 cells were treated with either IFN/LPS or PBS at the time of weekly media change. Aqueous spermine nonoate was then added to give the final indicated concentration. After one week, media samples were analyzed for 8-epi-prostaglandin F2α as described in the Methods section. Values are reported as pg/ml 8-epi-prostaglandin F2α ± SEM (n = 6).

**Figure 5:**
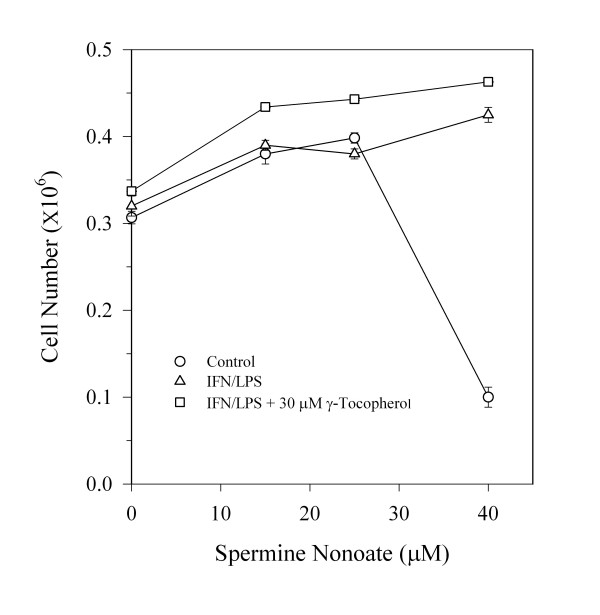
Effect of IFN/LPS treatment on the toxicity of spermine nonoate. Cell counts on cultures treated as described in Figure 4 were conducted utilizing a Coulter Counter (see Methods section). In addition one set of cell cultures was treated with 30 μM γ-tocopherol. Data are reported as the mean # of cells per dish ± SEM (n = 6).

To determine if induction of NO causes cellular lipid oxidation, levels of nitrite and 8-epi-prostaglandin F2α (a radical-mediated oxidation product of arachidonic acid) were determined in media, as shown in Figure 3 in response to IFN/LPS ± PBIT treatment. PBIT effectively blocked both nitrite and 8-epi-prostaglandin F2α formation in confluent C3H 10T1/2 cells, consistent with NO-mediated intracellular generation of isoprostane from arachidonic acid and in agreement with the observed protective effect of PBIT on cellular tocopherol levels. Combination treatment with alpha + gamma -tocopherols significantly decreased basal isoprostane formation, but enhanced the effect of IFN/LPS treatment (Figure 3). To further demonstrate that NO mediates isoprostane formation, the effect of increasing concentrations of spermine nonoate (exogenous NO generator) on cellular isoprostane formation was determined, both in the presence and absence of endogenous NO that was induced by IFN/LPS treatment (Figure 4). Consistent with the data shown in Figure 3, exogenous NO caused a progressive increase in media isoprostane levels, for treatment levels up to 30 μM spermine nonoate. Interestingly a corresponding cytotoxic effect was noted at concentrations of spermine nonoate above 30 μM. However, cells treated with IFN/LPS or γ-tocopherol prior to spermine nonoate treatment were somewhat protected from the toxic effects of 30 μM spermine nonoate, despite the significantly greater NO exposure for these cells (Figure 5). At levels higher than 40 μM, spermine nonoate was toxic to all treatment groups (data not shown).

### Oxidation products of tocopherols in tissue culture

Among the tocopherols, γ-tocopherol is observed to react preferentially with NO_2 _in a lipophilic model system such that when a mixture of the 4 tocopherol analogues is exposed to NO_2_, only γ-tocopherol reacts until it disappears, followed by the subsequent destruction of the other tocopherol analogues [44]. γ-Tocopherol has previously been reported to react with NO_2 _to form two main oxidation products (5-nitro-γ-tocopherol and the ortho-quinone, tocored) *in vitro *depending upon the polarity of the reaction environment [12]. In a polar aqueous environment, 5-nitro-γ-tocopherol is formed preferentially, whereas in a lipid phase environment, tocored is generated [12]. In order to determine the nature of tocopherol reaction products formed in cells growing in culture after IFN/LPS treatment, cultures containing either 10 μM α-tocopherol or 10 μM γ-tocopherol were exposed to IFN/LPS for 7 days and reaction products were determined. IFN/LPS treatment significantly increased media and cellular levels of α-tocopherol quinone (Table 2), however, no significant increase in reaction products of γ-tocopherol, including 5-nitro-γ-tocopherol, tocored, or γ-tocopheryl-quinone was detected in media. However a 39% increase in γ-tocopheryl-quinone in cells was noted (Table 2).

**Table 2:** Enhanced formation of α-tocopheryl-quinone in medium and cells of IFN/LPS-treated C3H 10T1/2 cells. Confluent cultures of C3H 10T1/2 cells were pre-treated for seven days as indicated with either 10 μM α-tocopherol, 10 μM γ-tocopherol, or with 25 μL ethanol as a control. The cell culture media was then changed and cultures treated with either PBS (control) or IFN/LPS for seven days. To another group of dishes treated with either tocopherol for 7 days, 100 μM of the NO donor, SIN-1, was given to cells for 2 hours. Samples of the media and cells were harvested and assayed for tocopherols and tocopherol oxidation products. Data are expressed as means ± SEM (n = 3).

	Tocopherol	Oxidant	Tocopherol nmoles/10^6 ^cells *(% of Control)*	Tocopheryl Quinone nmoles/10^6 ^cells *(% of Control)*	5-Nitro-γ-Tocopherol nmoles/10^6 ^cells
Cells	α-Tocopherol	Control	1.32 ± 0.06	0.056 ± 0.002	-
	α-Tocopherol	IFN/LPS	1.42 ± 0.15 *(+7.5%)*	0.072 ± 0.005 (+28.6%)**	-
	α-Tocopherol	SIN-1	1.38 ± 0.02 *(+4.5%)*	0.290 ± 0.008 (+418%)*	-
	γ-Tocopherol	Control	1.49 ± 0.07	0.0067 ± 0.0008	nd
	γ-Tocopherol	IFN/LPS	2.50 ± 0.12 (+67.8%)*	0.0093 ± 0.0004 (+38.8%)**	nd
	γ-Tocopherol	SIN-1	1.50 ± 0.05 *(+0.7 %)*	0.01 ± 0.005 (+49.2%)**	nd

			nmoles/dish	nmoles/dish	nmoles/dish

Media	α-Tocopherol	Control	47.8 ± 2.0	0.79 ± 0. 08	-
	α-Tocopherol	IFN/LPS	35.6 ± 1.1 (-25.5%)**	1.66 ± 0.06 (+110%)*	-
	α-Tocopherol	SIN-1	1.9 ± 0.2 (-96%)*	34.0 ± 1.1 (+4,203%)*	-
	γ-Tocopherol	Control	50.6 ± 1.4	0.30 ± 0.1	nd
	γ-Tocopherol	IFN/LPS	49.7 ± 1.3 *(-1.8%)*	0.28 ± 0.05 *(-6.6%)*	nd
	γ-Tocopherol	SIN-1	1.6 ± 0.5 (-96.7%)*	1.50 ± 0.02 (+400%)*	11.9 ± 0.3*

Significantly different from its corresponding control value * p < 0.01; ** p < 0.05 nd = not detected

Although α-tocopherol levels dropped approximately 30% in IFN/LPS-treated cultures, no decrease in media γ-tocopherol levels was seen relative to control cultures (Table 2). This is in contrast to what was observed in Figure 1 when both α- and γ-tocopherols were present simultaneously and each decreased approximately 15% with IFN/LPS treatment relative to controls. This suggests the possibility of a reaction between an oxidized α-tocopherol intermediate and γ-tocopherol in the media, potentially sparing α-tocopherol at the expense of the γ-analogue, analogous to the regeneration of α-tocopherol from its radical intermediate by ascorbate [45]. Exposure of tocopherol-containing cell cultures to the NO generator, SIN-1, destroyed > 90% of media tocopherol, yet did not significantly alter tocopherol levels in cells after two hours of exposure (Table 2). In γ-tocopherol-treated cell cultures exposed to SIN-1, 5-nitro-γ-tocopherol was the predominant product formed along with an unknown compound co-eluting with tocored, whereas α-tocopheryl-quinone predominated in α-tocopherol containing cultures exposed to SIN-1. Similar to experiments in which both α- and γ-tocopherols were simultaneously present (Figure 2), cellular γ-tocopherol increased significantly in cells in response to IFN/LPS treatment, whereas α-tocopherol remained unchanged from control values (Table 2).

## Discussion

The cellular response to infection is well known to involve oxidative attack by host cells on an invading organism. While the corresponding inflammatory state promotes clearance of the infection, it also is associated with cellular damage to the host cells and increased risk for the subsequent development of cancer [35,46]. The precise mechanism by which normal cells defend against the oxidative assault they release is less clearly understood. The results presented here suggest that host cellular defense responses may be initiated by the sequence of events mimicking a bacterial infection such that cells are protected to a greater extent from the effects of nitrogen oxidants. While the induction of NO synthesis is one key change that occurs in this C3H 10T1/2 cell line in response to IFN/LPS exposure, it appears that NO is responsible for only a small fraction of the loss of extracellular tocopherol that ensues (Figure 2). Presumably other oxidants are responsible for the loss of tocopherol in the media and these oxidants may preferentially react with α-tocopherol, as has been shown previously for the reaction of α-tocopherol with reactive oxygen species [25-27] and has been observed *in vitro *where α-tocopherol appears to react preferentially with extracellularly generated oxidants [47]. This would be consistent with the observed formation of the oxidized quinone form of the tocopherols and the absence of the nitro derivatives of γ-tocopherol in the medium of IFN/LPS-treated cells. The loss of γ-tocopherol in media only in the presence of α-tocopherol (Figure 2 vs Table 2), suggests that an interaction may occur between these two molecules, possibly through the reaction of the α-tocopheryl radical intermediate with γ-tocopherol, thereby reducing the oxidative loss of α-tocopherol at the expense of γ-tocopherol. When α-tocopherol alone was present in the media, the relative loss of α-tocopherol was doubled, yet when γ-tocopherol alone was present, no loss of tocopherol in the media was observed. It is interesting to note that measurement of media tocopherol, cellular tocopherol and known oxidation products only account for approximately 70–80% of the original tocopherol with which cells are treated. This is in agreement with the results reported by Gao, et al. [22] in macrophages treated with tocopherol, where approximately 26% of α-tocopherol was unaccounted for either as tocopherol or its known oxidation products. Gao et al. [22] also reported that γ-tocopherol significantly enhanced uptake of α-tocopherol in macrophages.

Despite the enhanced generation of oxidants when cells are treated with IFN/LPS, cellular levels of γ-tocopherol paradoxically increase significantly and α-tocopherol remains constant or decreases only slightly. This is consistent with the observations of Jiang et al [48], who, utilizing a rat model of inflammation, observed no effect on α-tocopherol and an increase in cellular and plasma γ-tocopherol concentrations *in vivo *in response to inflammation, along with a reduction in protein nitration with γ-tocopherol supplementation. It is not clear what the mechanism(s) responsible for increased levels of cellular tocopherol resulting from IFN/LPS treatment may be. Possibilities include enhanced cellular uptake/decreased cellular export, decreased degradation, or the conversion of tocopherol from a storage form not observed in the analysis of free tocopherol. In the first case a transport system would theoretically be required which could be controlled in some manner to cause increased uptake of tocopherol under inflammatory conditions or decreased cellular excretion. From our studies we see no evidence for such a selective uptake system, as cellular levels generally appear correlated with external concentrations. The second possibility, that degradation is decreased, would appear to be counterintuitive given the increase in oxidation products observed for both alpha and gamma tocopherols when cells were treated with IFN/LPS, however, a mechanism involving both increased transport of tocopherols into cells and enhanced degradation of α-tocopherol would fit with the experimental data. A third possibility to explain the elevation of cellular tocopherol in response to inflammation is that tocopherols may exist in storage forms. The recent discovery of α-tocopheryl phosphate [49] provides a potential candidate for such a storage system in cells. Negis et al. [50] report that approximately 13% of α-tocopherol in rabbit serum exists as α-tocopheryl phosphate and that it represents a more bioactive form of tocopherol. Conversion of this form of tocopherol to the lipid soluble form in response to IFN/LPS could explain the curious increase in tocopherol observed under conditions of increased oxidative stress and could conceivably be under the control of a cellular phosphatase, however, it is not yet known if γ-tocopherol forms such a molecule. Other possible storage forms could include esters such as the acetate or glucoronides. It is also possible that intracellularly, α-tocopherol spares γ-tocopherol by reacting with a γ-tocopheryl intermediate, however, this would not explain the overall increase in γ-tocopherol observed, as cellular γ-tocopherol levels increased similarly both in the presence and absence of α-tocopherol (Figure 2 and Table 2). The conditions utilized in the present study assess only the steady state levels of tocopherols and their oxidation products and, therefore, may not capture kinetic changes that occur. However, the results are qualitatively similar to those reported by Gao, et al. [22], who did study kinetic changes in cellular levels of tocopherols and their oxidation products.

In contrast to the minimal effects of NO inhibition on the loss of media tocopherol, intracellular concentrations of both α- and γ-tocopherols are significantly increased through the inhibition of NO synthesis by PBIT (Figure 2), suggesting that intracellular loss of tocopherol is largely mediated by NO and/or its reaction products. NO-mediated oxidation of key cellular targets was also observed in these cells as evidenced by the formation of the oxidative marker, 8-epi-prostaglandin F2α (Figures 3 and 4), and an increase in DNA strand breaks previously described for IFN/LPS-treated 10T1/2 cells [44]. Interestingly, it was previously observed that α-tocopherol alone was ineffective at reducing either isoprostane formation or DNA damage in IFN/LPS-stimulated cells, whereas the presence of γ-tocopherol, β-tocopherol, or δ-tocopherol alone reduced both types of oxidative damage to near control levels [44]. This is consistent with previous reports of the comparative effects of tocopherols on NO_2_-mediated damage in which a similar superiority of γ-tocopherol over α-tocopherol was demonstrated in mammalian cells [10,13,51]. The apparent inability of α-tocopherol and γ-tocopherol to reduce isoprostane levels when both are simultaneously present (Figure 3) is intriguing and requires further study. It would appear from the data presented here that isoprostane formation is mediated by NO, probably through its oxidation products, such as NO_2 _and/or peroxynitrite. This is consistent with the report of Marnett, et al. [52] who showed that deletion of the iNOS gene significantly decreased F2 isoprostane formation *in vitro *and *in vivo *in response to IFN/LPS treatment. Ramsey et al [53] also demonstrated that isoprostane formation induced by Chlamydia infection was mediated through iNOS as well. The absence of known markers of NO_2 _or peroxynitrite-mediated oxidation of γ-tocopherol suggests either metabolism of the products or that another oxidative intermediate may be involved.

The types of oxidized tocopherols that are observed in cell culture can indicate the nature of the oxidants that are being generated. The absence of 5-nitro-γ-tocopherol in cells treated with IFN/LPS (Table 2) suggests the absence of nitrating species such as NO_2 _and peroxynitrite in the aqueous phase of IFN/LPS treated cultures (or its metabolism). In the case of SIN-1 treated cells, such a reaction involving a nitrating species occurs to a significant extent in the medium as evidenced by the formation of 5-nitro-γ-tocopherol as the principal reaction product (Table 2), consistent with the results described by Hoglen et al [54]. This suggests that the chemical oxidants generated by SIN-1 are not representative of those produced and released extracellularly by living cells stimulated with cytokines. The data presented suggests a model in which extracellular oxidants are generated by cells and that these oxidants preferentially react with α-tocopherol to form α-tocopherol oxidation products, principally α-tocopheryl-quinone. Intracellularly, NO is generated, possibly as an anti-oxidant [55,56], and any intracellular reactive oxygen or reactive nitrogen species that form as a byproduct may be detoxified by α-tocopherol and γ-tocopherol, respectively. The absence of 5-nitro-γ-tocopherol and tocored in NO producing cells suggests that peroxynitrite and NO_2 _may not be formed intracellularly to a significant extent under the conditions used in these experiments, despite its potentially enhanced rate of formation from the oxidation of NO in lipid phase environments [32]. Alternatively it is possible that γ-tocopherol reaction products may be further metabolized in cells, for example by a nitratase enzyme [57] and are therefore not detected. Increased γ-tocopheryl-quinone in cells treated with IFN/LPS, but not in the media, confirms that γ-tocopherol does indeed react with some oxidative specie(s) within the cell, but not in the media. The generation of a product distinct from that observed previously for the reaction of γ-tocopherol with reactive nitrogen species, yet similar chromatographically to tocored, was also observed and suggests a possible unique reaction pathway. One likely explanation is that low oxygen tension within the cells does not favor the complete conversion of NO to NO_2_, but rather forms the peroxynitrite radical intermediate, which then reacts preferentially with γ-tocopherol to form γ-tocopheryl quinone, as distinguished from the reaction products seen for other oxidative nitrogen species [12]. Such a transient intermediate was observed previously and postulated as a potential agent responsible for the DNA damaging effects of NO in cells [58] in which oxidation of NO was essential for cellular DNA damage, but NO_2 _was determined not to be the agent responsible.

## Conclusion

Cytokine-mediated inflammation is clearly an important component in an organism's defense against infections, however, it is a double-edged sword that may also play a significant role in aging-related diseases. Optimizing immune function while minimizing damage to normal tissue may be key to reducing premature death from aging-related diseases. The observed increase in cellular tocopherol concentrations may be an important component of the cell's response to infection and appears to be specifically associated with mitigating damage resulting from cellular NO synthesis. The role of tocopherols in the process of cell-mediated immunity remains to be fully delineated, however, studies elucidating the specific biological and chemical mechanisms of action for the tocopherols may ultimately lead to a better understanding of their value in the prevention and cure of acute and chronic diseases.

## Methods

### Chemicals

RRR-α-Tocopherol was purchased from Fluka Biochemika (Ronkonkoma, NY). RRR γ-tocopherol was purchased from Sigma Chemical Co. (St. Louis, MO). Tocol (3,4-dihydro-2-methyl-2-(4,8,12-trimethyltridecyl)-6-chromanol) was used as an internal standard in chromatography and was a gift from Hoffmann-LaRoche (Basel, Switzerland). The concentrations of the tocopherol stock solutions were determined by optical density readings at the indicated wavelength with maximum absorption (λ_max_) after diluting the stock solutions of the tocopherols to the appropriate concentration with ethanol: α-tocopherol (λ_max_= 292 nm; E^1% ^= 75.8); γ-tocopherol (λ_max_= 298 nm; E^1% ^= 92.8)

Murine IFN-γ was obtained from Life Technologies, Inc., Grand Island, NY. Bacterial LPS (Escherichia coli, serotype 0127:B8) and 3-morpholino-sydnonimine (SIN-1) were from Sigma Chemical Co., St. Louis, MO. S, S'-1,4-Phenylene-bis(1,2-ethanedieyl)bis-thiourea dihydrobromide (PBIT) and 8-epi-prostaglandin F2α standard were purchased from Cayman Chemical Co., Ann Arbor, MI. Spermine nonoate was obtained from Calbiochem, San Diego, CA.

### Cell culture

C3H 10T1/2 mouse fibroblasts (ATCC no. CCL 226), passages 8–15, were used for all experiments [59]. Unless otherwise stated, cells were seeded in plastic tissue culture dishes at a density of 4000 cells/ml in Eagle's basal medium (BME) supplemented with 5% bovine calf serum (Fisher Scientific, Hampton, NH.) and gentamycin sulfate (25 μg/ml), and incubated at 37°C in a humidified atmosphere of 5% CO_2_/95% air. At this seeding density, confluent monolayers are normally formed by day 6 or 7 after plating. Cell numbers were determined as follows: Cells were prepared by aspirating the media, washing the cells with PBS and versene (0.2% ethylene diamine tetraacetic acid in PBS), followed by trypsinization (0.4% trypsin in PBS) in culture dishes for two minutes. Cells were collected into sterile culture tubes followed by dilution with fresh media to inhibit trypsin and cell counts performed using a Coulter counter (Coulter Electronics; Hialeah, FL).

### Mixed tocopherol treatment of C3H 10T1/2 cells

Treatments were done in triplicate. C3H 10T1/2 mouse fibroblast cells were grown to confluence in 100 mm dishes (N = 12) with 10 ml of culture medium. At confluence, a mixture of α- & γ-tocopherol (10 μM, final concentration for each) in ethanol (50 μl) was added to each dish. After seven days cell culture medium was changed and cells were re-treated with tocopherol. At this time six of the 12 dishes were also treated with IFN (10 ng/ml) & LPS (1 μg/ml) to stimulate NO production. Three culture plates that were treated with IFN/LPS and three that contained only the mixture of tocopherols, were also treated with 50 μl of 2 mM PBIT, a selective iNOS inhibitor, in phosphate-buffered saline (PBS) yielding a final media concentration of 10 μM PBIT. The other 6 dishes were treated with either 50 μl PBS vehicle or PBIT (10 μM final concentration). Seven days later, media nitrite and 8-epi-prostaglandin F2α levels were measured according to the methods described below in duplicate for each culture dish. Media samples (1.0 ml for nitrite and 100 μl for 8-epi-prostaglandin F2α analyses) from each culture dish were removed and stored frozen in amber vials (12 × 32 mm, screw cap). Cell samples were prepared for analysis by aspirating the media, washing the cells with PBS and versene (10 μM), followed by trypsinization for 3 minutes. Using a rubber policeman, the dishes were scraped to ensure that all the cells lifted off from the plate. The cells were given fresh media (10 ml) to inhibit the trypsin, and then pipetted into 15 ml centrifuge tubes. Cell numbers were determined by taking 1 ml of the media and diluted with 19 ml of 0.9% NaCl, and counted using a Coulter counter. The remaining volume in tubes was centrifuged for 15 min at 2500 rpm. After centrifuging, media was again aspirated and PBS (2 ml) added to the cells and vortexed until they resuspended into the solution. Solution was then pipetted into Kimax culture tubes with caps. To assure the transfer of all cells, the centrifuge tube was rinsed with PBS (2 ml), which was added into the culture tubes. The cell suspension was then centrifuged for 15 min at 2500 rpm. After 15 min the supernatant was aspirated and 1 ml PBS was added to cells, and all samples were stored in a freezer @ -20°C. Tocopherol levels in both the media and cells were determined as described below.

### Extraction of tocopherols

#### Media

At the time of extraction, media (1 ml) samples were thawed and mixed with ethanol (1 ml), followed by addition of hexane (1 ml). The mixture was then vortexed for one minute and centrifuged for five minutes at 2500 rpm. The hexane phase containing the tocopherol extract was transferred into an amber vial and dried under nitrogen gas. This process was repeated three times for each media sample. The dried extract was then dissolved into 200 μl of 100% acetonitrile and analyzed by High Pressure Liquid Chromatography (HPLC) as described below.

#### Cells

Cellular samples were defrosted and diluted with 300 μl of pronase, then incubated in a water bath at 37°C. After 30 min, 500 μl of sodium dodecylsulfate (SDS) and 1 ml of ethanol was added to each sample. Tocopherols from the cells were extracted three times with 1 ml of hexane. The sample extract was then transferred into an amber vial and dried under nitrogen, as described previously for media. The dried cell extract was then dissolved in 50 μl of acetonitrile for analysis by HPLC.

### Analytical methods

#### Tocopherols

Tocopherols were analyzed by HPLC according to the method of Cooney et al [12]. A Waters Novapak C-18 reverse-phase (RP) column (10 × 4.6 mm; 5 μm particle size) eluted with 100% acetonitrile was used at a flow rate of 1.0 ml/min. The compounds were measured simultaneously at 270 nm and 295 nm using a Beckman HPLC equipped with a dual wavelength diode array detector.

#### Tocopherol oxidation products

Altered products of γ- or α-tocopherols in cell cultures were measured as follows. Cells were grown to confluence in 100 mm dishes. After a media change, cells were treated with either ethanol vehicle (25 μl), α-tocopherol, or γ-tocopherol at a final concentration of 10 μM. One week later at the time of media change cells were retreated with tocopherols and within each tocopherol treatment group, a subset of dishes was also treated with either PBS as a control or IFN (10 ng/ml) and LPS (1 μg/ml). To a separate group of cells treated with tocopherols only, the NO donor, 3-morpholino-sydnonimine (SIN-1) dissolved in PBS was added to the cell culture medium (final concentration – 100 μM) seven days after the final tocopherol treatment and media change. After a SIN-1 treatment period of two hours, media and cellular samples from all treatment groups (including IFN/LPS-treated and tocopherols only) were collected to determine the presence of altered tocopherol products. Tocopherol extraction with hexane was performed and altered tocopherol products in these fractions were then analyzed by reverse-phase HPLC as described above. Three independent experiments were performed to measure the effects of the various forms of tocopherol + IFN/LPS for each tocopherol and treatment group.

#### 8-epi-prostaglandin F2a

As a measure of cellular oxidation caused by IFN/LPS treatment, media levels of 8-epi-prostaglandin F2α were measured in 100 μl samples of cell culture media using an enzyme immunoassay kit (#516351) from Cayman Chemical Co., Ann Arbor, MI according to the directions of the manufacturer. A standard curve utilizing authentic 8-epi-prostaglandin F2α standard was generated for each assay and values for 8-epi-prostaglandin F2α are reported as pg/ml of media.

#### Nitrite analysis

The concentration of nitrite in culture media was determined by a modification of the Saltzman assay [60]. Briefly, 100 μl aliquots of media were mixed with 900 μl of absorbing reagent (0.5% sulfanilic acid, 0.002% *N*-1-naphtylethylenediamine dihydrochloride, 14% glacial acetic acid) and incubated at room temperature for 30 min. Light absorbance (546 nm) of the solution was then measured in a Shimadzu UV160U spectrophotometer and compared to standard solutions of sodium nitrite dissolved in distilled water and added to the absorbing reagent. The detection of nitrite with this assay was linear over the range of 0.2 μM to at least 30 μM. All experimental values were corrected for the nitrite content of time-matched culture media and incubation buffers devoid of cells.

### Statistical analysis

Treatment groups were analyzed by One Way Analysis of Variance (ANOVA) utilizing GraphPad Software Inc. (San Diego, CA) InStat program. A Dunnett multiple comparisons test was used to determine significant differences between treatment groups and their respective control. Deviations from normality in standard deviations were tested by the method of Bartlett. In those cases where there were significant differences in standard deviations between groups, values were log-transformed prior to statistical analysis. Comparisons between control and single treatment or between media vs cellular mean values were analyzed by a two-tailed unpaired student t-test.

## Abreviations

IFN – *interferon-γ*

LPS – *bacterial lipopolysaccharide*

NO – *nitric oxide*

nd – *not detected*

NO_2 _– *nitrogen dioxide radical*

PBIT – *S, S'-1,4-Phenylene-bis(1,2-ethanedieyl)bis-thiourea dihydrobromide*

iNOS – *inducible nitric oxide synthase*

PBS – *phosphate-buffered saline*

HPLC – *high pressure liquid chromatography*

SIN-1 – *3-morpholino-sydnonimine*

SEM – *standard error of the mean*

## Authors' contributions

YT conducted the uptake studies, the oxidation product studies, all HPLC analysis, supervised Pamela Utu in the data collection for Figure 2, and drafted the methods section and results section first drafts; LALW supervised Alison Motosue to obtain the data on NO toxicity and provided assistance with the writing and editing of the manuscript; RVC conceived and designed the study, supervised YT and LALW on all aspects of the study, in addition to supervising Leila Okinaka in the isoprostane analysis, RVC was responsible for writing the balance of the paper and its revision. All authors reviewed and approved the final manuscript.
